# Performance of predictive scores for in-hospital mortality in surgical infective endocarditis: a retrospective cohort study

**DOI:** 10.3389/fcvm.2026.1829289

**Published:** 2026-05-26

**Authors:** Paula Ximena Pavia-Velandia, Paula Liliana Torres-Gómez, Nicolás Molano-González, Adelaida Rodríguez-Villegas, Tomás Castillo-Valderrama, Fabián Andrés Guaque-Camargo, Sara Mariana Hidalgo-Suárez, Carlos Morales-Pertus, David René Rodríguez-Lima

**Affiliations:** 1 Institute of Infectious Diseases and Tropical Medicine, Méderi Research Center (CIMED), Hospital Universitario Mayor Méderi, Universidad del Rosario, Bogotá, Colombia; 2 Cardiovascular Surgery, Hospital Universitario Mayor- Méderi, Bogotá, Colombia; 3 Clinical Research Group, School of Medicine and Health Sciences, Universidad del Rosario, Bogotá, Colombia; 4 School of Medicine and Health Sciences, Universidad del Rosario, Bogotá, Colombia; 5 Cardiac and Thoracic Institute, Méderi Research Center (CIMED), Hospital Universitario Mayor Méderi, Universidad del Rosario, Bogotá, Colombia

**Keywords:** calibration, cardiac surgery, infective endocarditis, Latin America, model validation, mortality prediction, risk scores

## Abstract

**Background:**

Infective endocarditis (IE) carries high surgical mortality, particularly in Latin American populations where validated prediction models are scarce. Although EuroSCORE II, RISK-E, and APORTEI have shown acceptable performance in European cohorts, external validation in middle-income country settings remains limited.

**Methods:**

We conducted a retrospective cohort study of adult patients who underwent cardiac surgery for IE at a high-complexity tertiary-care center in Bogotá, Colombia, between 2014 and 2025. The primary outcome was all-cause in-hospital mortality. Discrimination was assessed using Harrell's C-statistic and calibration was assessed via intercept, slope, Spiegelhalter's z-test, Brier score, mean absolute error (Eavg), and maximum absolute error (Emax).

**Results:**

Seventy patients were included (mean age 51.9 ± 14.8 years; 70% male); in-hospital mortality was 30% (21/70). RISK-E demonstrated the highest discrimination (C = 0.741, 95% CI 0.61–0.85), followed by EuroSCORE II (C = 0.718, 95% CI 0.58–0.83) and APORTEI (C = 0.676, 95% CI 0.53–0.80). All three scores systematically underestimated mortality risk, as evidenced by positive calibration intercepts (range 1.14–1.75). Calibration was closest to ideal for RISK-E (slope=0.962), while APORTEI showed the greatest deviation (slope=1.425). Spiegelhalter's z-test indicated significant miscalibration for all three models (all *p* < 0.001).

**Conclusions:**

RISK-E showed numerically superior discrimination and a calibration slope closest to unity, although pairwise DeLong's tests showed no statistically significant difference between any two scores, and these findings should be interpreted in the context of the limited sample size (70 patients, 21 events). All three scores systematically underestimated in-hospital mortality, suggesting that recalibration or development of locally derived models is needed for reliable risk stratification in Latin American IE surgical programs.

## Background

Infective endocarditis (IE) remains a life-threatening condition with substantial in-hospital mortality. Globally, reported mortality ranges from 15% to 30% (1); in Latin America rates approach 33% (95% CI 28%–38%) (2), and in Colombia, mortality has been reported at up to 39% (3). Despite advances in antimicrobial therapy and surgical technique, these rates have remained largely unchanged over recent decades, driven by an increasing burden of prosthetic valve and healthcare-associated infections, an ageing population, and growing immunocompromised patient populations (4, 5).

Approximately 25%–50% of IE patients require cardiac surgery during the acute phase (6). Accurate preoperative risk stratification is essential to inform shared decision-making, optimize timing of surgery, and benchmark institutional outcomes. EuroSCORE II, the most widely used general cardiac surgery risk model, was developed in a population where only 2.2% of patients had active endocarditis and elective procedures predominated (76.7%), limiting its applicability to surgical IE (7). IE-specific scores such as RISK-E and APORTEI incorporate endocarditis-relevant variables, including causative microorganism, periannular complications, and sepsis, and have demonstrated superior predictive performance over general models in European and North American cohorts (8–12).

At our institution, EuroSCORE II is the model routinely used for risk stratification in IE patients; however, preliminary analyses showed that while EuroSCORE II values were higher in non-survivors, this difference did not reach statistical significance, suggesting suboptimal model fit in our population. No external validation of RISK-E or APORTEI has been reported in a Colombian or broadly Latin American surgical IE cohort. Therefore, the aim of this study was to assess and compare the predictive performance of RISK-E, EuroSCORE II, and APORTEI for in-hospital mortality in patients with IE who underwent cardiac surgery at a high-complexity hospital in Bogotá, Colombia.

## Methods

### Study design and population

**Figure 1:**
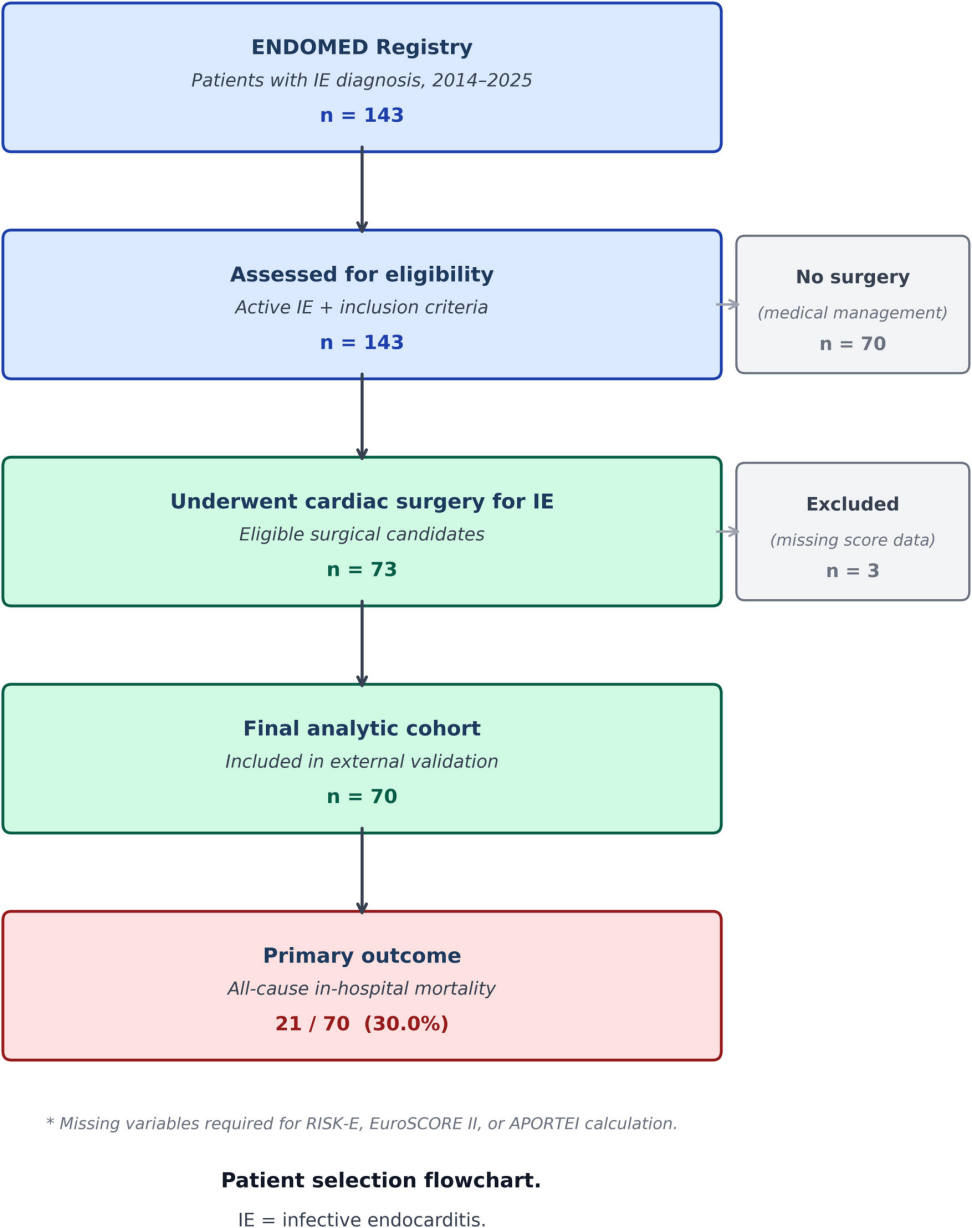
Patient selection flowchart. Of 143 patients with infective endocarditis documented in the ENDOMED registry between January 2014 and December 2025, 73 (51.0%) underwent cardiac surgery and were eligible for inclusion. Three patients (4.1%) were excluded due to missing variables required for score calculation. The final analytic cohort comprised 70 patients.

We conducted a retrospective cohort study at a high-complexity tertiary-care hospital in Bogotá, Colombia. Adult patients (≥18 years) diagnosed with IE according to the modified Duke criteria who underwent cardiac surgery between January 2014 and December 2025 were eligible for inclusion. Patients were excluded if data required to calculate any of the three risk scores were missing, or if the surgical indication was unrelated to active IE. Of 143 patients registered in the IE clinical registry (ENDOMED registry) during the study period, 73 (51.0%) underwent cardiac surgery and met the eligibility criteria; 3 patients (4.1%) were subsequently excluded due to missing variables required for score calculation, yielding a final analytic cohort of 70 patients (Figure 1). Given the low exclusion rate (4.1%), a complete-case analysis was deemed appropriate, as the excluded patients would not materially affect the reported performance metrics. The primary outcome was all-cause in-hospital mortality during the index hospitalization.

### Data collection

Clinical, demographic, microbiological, echocardiographic, and surgical data were obtained from the institutional IE clinical registry (ENDOMED registry), which consolidates structured data from electronic medical records, laboratory databases, and diagnostic imaging systems. All records were fully anonymized prior to analysis.

### Risk score calculation

EuroSCORE II, RISK-E, and APORTEI were calculated for each patient using the variables specified in their respective original publications. EuroSCORE II was computed using the official online calculator. RISK-E and APORTEI were calculated from registry variables collected at the time of surgical evaluation. All three scores were specifically assessed using data available at the time of preoperative surgical evaluation, immediately prior to the index surgical procedure. For APORTEI, variables were collected in their original categorized form as specified in the score's development study. Missing data for score components were not imputed; patients with incomplete score variables were excluded from the analysis of the affected score.

### Statistical analysis

Descriptive statistics are reported as mean (standard deviation) or median (interquartile range) for continuous variables, and as frequencies and percentages for categorical variables. Between-group comparisons used the independent samples t-test or Mann–Whitney U test for continuous variables and the chi-square or Fisher's exact test for categorical variables, as appropriate. In an exploratory analysis, univariate logistic regression models were fitted for each candidate variable in relation to in-hospital mortality; unadjusted odds ratios (OR) with 95% confidence intervals are reported.

Risk score performance was evaluated through discrimination and calibration. Discrimination was quantified using Harrell's C-statistic, equivalent to the area under the receiver operating characteristic (ROC) curve, with 95% confidence intervals estimated by 2,000-resample stratified bootstrap (pROC package). Calibration was assessed through the calibration intercept and slope, where values of 0 and 1 indicate perfect calibration, respectively. Miscalibration was formally tested using the U-statistic and Spiegelhalter's z-test. Global model accuracy was quantified using the Brier score, mean absolute error (Eavg), and maximum absolute error (Emax). Pairwise differences in discrimination between scores were formally compared using DeLong's test for correlated ROC curves (pROC package).

All analyses were performed using R (version 4.4; R Foundation for Statistical Computing, Vienna, Austria) with the rms package and the pROC package for confidence interval estimation (13).

## Results

### Study population and clinical outcomes

A total of 70 patients met the inclusion criteria and underwent cardiac surgery for IE during the study period (see Figure 1 for the patient selection flowchart). In-hospital mortality was 30% (21/70). Non-survivors were significantly older than survivors (mean 57.81 ± 14.78 vs. 49.43 ± 14.17 years; *p* = 0.02). Non-survivors also had significantly lower baseline hematocrit compared to survivors (32.39 ± 4.88% vs. 35.66 ± 7.03%; *p* = 0.03). No other statistically significant differences were observed in sociodemographic characteristics or baseline clinical conditions between groups (Table 1).

**Table 1:** Sociodemographic, baseline characteristics, and outcomes of patients undergoing cardiac surgery for infective endocarditis (*n* = 70).

Variable	Total (*n* = 70)	Survivors (*n* = 49)	Non-Survivors (*n* = 21)	*p*-value
Age, mean (SD)	51.94 (14.76)	49.43 (14.17)	57.81 (14.78)	0.02
Male sex, *n* (%)	49 (70%)	36 (73%)	13 (62%)	0.49
Coexisting Conditions and Risk Factors				
Hypertension	23 (33%)	13 (27%)	10 (47%)	0.15
Previous cardiac surgery	12 (17%)	10 (20%)	2 (10%)	0.33
Chronic kidney disease	10 (14%)	5 (10%)	5 (24%)	0.26
Diabetes mellitus type 2	8 (11%)	6 (12%)	2 (10%)	1.00
Smoker	10 (14%)	6 (12%)	4 (19%)	0.47
Laboratory Results				
Hematocrit, mean (SD)	34.68 (6.60)	35.66 (7.03)	32.39 (4.88)	0.03
Leucocytes, median (IQR)	7,210 (1,612–9,585)	6,930 (1,578–9,120)	7,770 (1,824–10,560)	0.31
Platelets ×10^3^, mean (SD)	277.8 (131.1)	283.7 (130.7)	264.0 (134.3)	0.56
Positive blood culture, *n* (%)	50 (71%)	34 (69%)	16 (76%)	0.77
Predictive Score Values				
EuroSCORE II, median (IQR)	7.25 (3.16–14.13)	4.37 (2.80–10.62)	11.45 (8.01–20.27)	0.004
RISK-E, median (IQR)	0.08 (0.06–0.15)	0.08 (0.03–0.12)	0.15 (0.08–0.22)	0.001
APORTEI ≥21 points, *n* (%)	9 (13%)	2 (4%)	7 (33%)	0.002
Surgical Characteristics				
Urgent surgery, *n* (%)	37 (53%)	25 (51%)	13 (62%)	0.31
Persistent infection (indication), *n* (%)	51 (73%)	38 (78%)	13 (62%)	0.24

IQR, interquartile range; SD, standard deviation; COPD, chronic obstructive pulmonary disease.

*P*-values from *t*-test or Mann–Whitney U for continuous variables and chi-square or Fisher's exact test for categorical variables.

Most patients underwent urgent or emergent surgery (53%), with persistent infection as the primary surgical indication. Median EuroSCORE II for the overall cohort was 7.25 (IQR 3.16–14.13). Non-survivors had significantly higher EuroSCORE II values than survivors (median 11.45 [IQR 8.01–20.27] vs. 4.37 [IQR 2.28–10.68]; *p* = 0.004). Similarly, APORTEI scores indicating higher risk (≥21 points) were more prevalent among non-survivors (7 vs. 2 patients; *p* = 0.002). RISK-E scores were also significantly elevated among non-survivors compared to survivors (median 0.15 [IQR 0.08–0.22] vs. 0.08 [IQR 0.03–0.12]; *p* = 0.001). No significant differences were observed between groups in ICU or in-hospital length of stay.

In the exploratory univariate logistic regression analysis, age (OR 1.04, 95% CI 1.03–1.06; *p* < 0.001) and hematocrit (OR 0.92, 95% CI 0.85–1.00; *p* = 0.046) were independently associated with in-hospital mortality. All three risk scores also demonstrated statistically significant associations: EuroSCORE II per unit (OR 1.09, 95% CI 1.02–1.16; *p* = 0.006), RISK-E per 0.01-unit increment (OR 1.06, 95% CI 1.01–1.12; *p* = 0.020), and APORTEI ≥21 points (OR 11.75, 95% CI 2.34–58.97; *p* = 0.002). Complete results are presented in Table 2.

**Table 2:** Univariate logistic regression analysis of predictors of in-hospital mortality in patients undergoing cardiac surgery for infective endocarditis (*n* = 70; 21 events).

Variable	OR (95% CI)	*p*-value	Events/N
Demographics and comorbidities			
Age (years)	1.04 (1.03–1.06)	**<0**.**001**	21/70
Male sex	0.59 (0.20–1.75)	0.338	21/70
Hypertension	2.52 (0.86–7.33)	0.091	21/70
Diabetes mellitus type 2	0.75 (0.14–4.08)	0.744	21/70
Chronic kidney disease	2.75 (0.70–10.82)	0.148	21/70
Previous cardiac surgery	0.41 (0.08–2.07)	0.281	21/70
Laboratory parameters			
Hematocrit (%)	0.92 (0.85–1.00)	**0**.**046**	21/70
Leukocytes (×10^3^/mm^3^)	1.06 (0.94–1.19)	0.342	21/70
Platelets (×10^3^/mm^3^)	1.00 (0.99–1.00)	0.565	21/70
Surgical characteristics			
Urgent surgery	1.28 (0.46–3.60)	0.639	21/70
Persistent infection (surgical indication)	0.47 (0.15–1.43)	0.183	21/70
Predictive risk scores			
EuroSCORE II (per unit increase)	1.09 (1.02–1.16)	**0**.**006**	21/70
RISK-E (per 0.01 unit increase)	1.06 (1.01–1.12)	**0**.**020**	21/66
APORTEI ≥21 points	11.75 (2.34–58.97)	**0**.**002**	21/70

OR, odds ratio; CI, confidence interval. Bold *p*-values indicate statistical significance (*p* < 0.05). All results from univariate logistic regression. RISK-E denominator *n* = 66 due to missing values in 4 records.

### Discrimination and calibration

**Figure 2:**
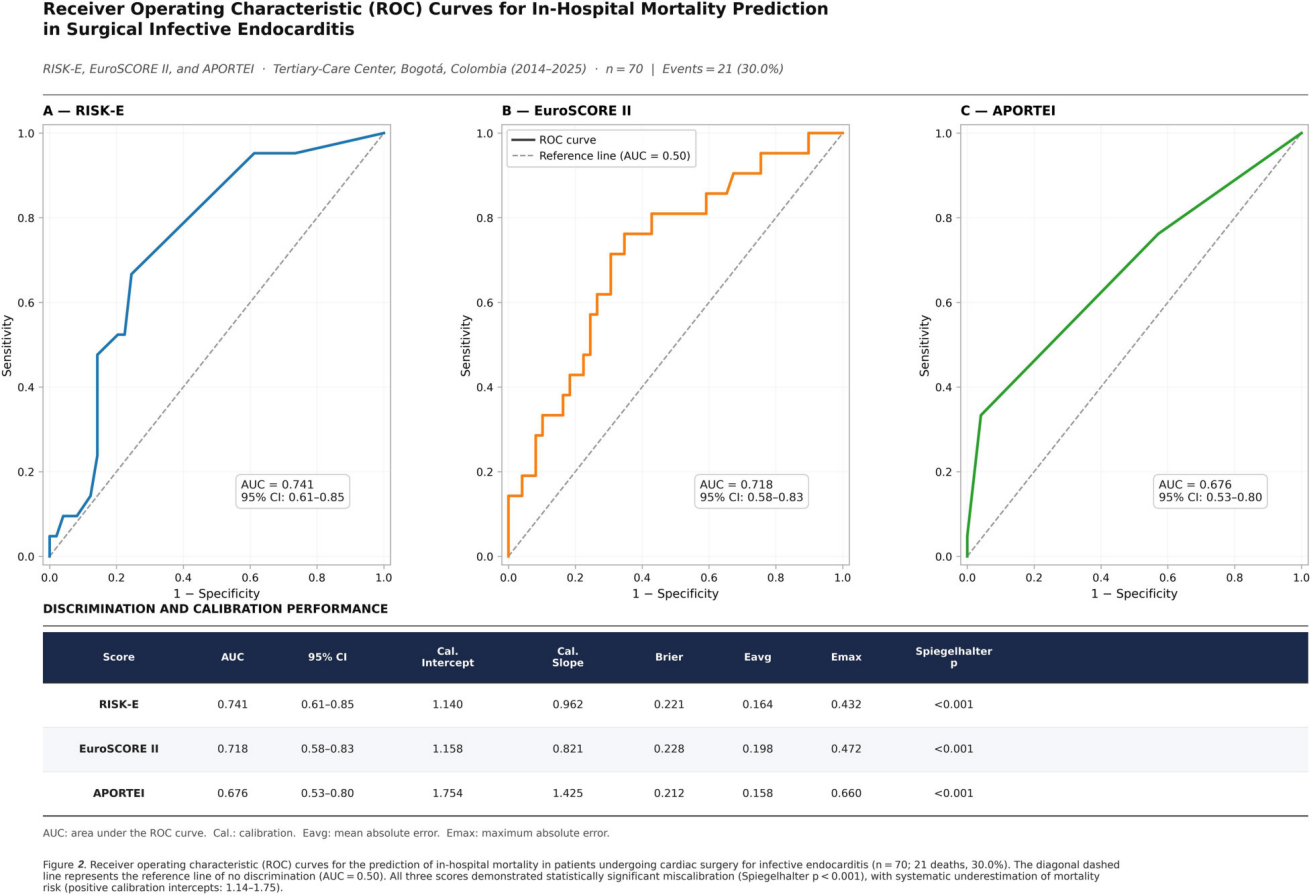
Receiver operating characteristic (ROC) curves for the prediction of in-hospital mortality in patients undergoing cardiac surgery for infective endocarditis (*n* = 70; 21 deaths, 30.0%). **(A)** RISK-E score (AUC=0.741, 95% CI 0.61–0.85); **(B)** EuroSCORE II (AUC=0.718, 95% CI 0.58–0.83); **(C)** APORTEI score (AUC=0.676, 95% CI 0.53–0.80). The diagonal dashed line represents the reference line of no discrimination (AUC=0.50). Discrimination and calibration metrics for all three scores are provided in the embedded table. All three scores demonstrated statistically significant miscalibration (Spiegelhalter *p* < 0.001), with systematic underestimation of mortality risk (positive calibration intercepts: 1.14–1.75).

The performance metrics for all three scores are presented in Figure 2. RISK-E demonstrated the highest discrimination (C = 0.741, 95% CI 0.61–0.85), followed by EuroSCORE II (C = 0.718, 95% CI 0.58–0.83) and APORTEI (C = 0.676, 95% CI 0.53–0.80). Pairwise DeLong's tests showed no statistically significant difference in AUC between any two scores (RISK-E vs. EuroSCORE II: *p* = 0.790; RISK-E vs. APORTEI: *p* = 0.344; EuroSCORE II vs. APORTEI: *p* = 0.696), indicating that the observed numerical differences in discrimination should be interpreted with caution.

All three models systematically underestimated absolute mortality risk in this cohort, as reflected by positive calibration intercepts (RISK-E: 1.14; EuroSCORE II: 1.16; APORTEI: 1.75). The calibration slope was closest to ideal for RISK-E (0.962), indicating appropriate weighting of predictors relative to observed outcomes. EuroSCORE II showed a slope below unity (0.821), suggesting overconfident risk predictions at the extremes of the distribution. APORTEI demonstrated the greatest calibration deviation (slope 1.425), likely reflecting the categorized collection of its component variables in this cohort, which attenuates the contribution of individual predictors.

Spiegelhalter's z-test indicated statistically significant miscalibration for all three models (all *p* < 0.001). The Brier score was lowest for APORTEI (0.212), followed by RISK-E (0.221) and EuroSCORE II (0.228). However, Emax was highest for APORTEI (0.661), indicating individual-level prediction errors of up to 66 percentage points for some patients. Eavg was comparable across models (RISK-E: 0.164; EuroSCORE II: 0.198; APORTEI: 0.158). ROC curves for each score are presented in Figure 2.

## Discussion

### Study population and risk score comparisons

IE remains a condition associated with substantial surgical mortality. In our cohort, in-hospital mortality was 30%, consistent with rates reported in Latin American series (33%, 95% CI 28%–38%) (2) and higher than the 15%–20% typically described in large contemporary European registries (14). This difference likely reflects referral patterns, delayed presentation, and resource constraints characteristic of middle-income healthcare settings.

The mean age of our cohort (51.94 ± 14.76 years) was notably lower than those in the development populations of EuroSCORE II (64.6 ± 12.5 years), APORTEI (63.6 ± 13.1 years), and RISK-E (61 ± 14 years) (7–9). This demographic difference is clinically relevant: younger IE patients in Latin America more frequently present with rheumatic valve disease, community-acquired infection, and different microbiological profiles compared to the predominantly prosthetic valve and healthcare-associated IE seen in European development cohorts. These differences may limit the transportability of scores derived from European populations to our setting.

Slightly more than half of our patients (53%) underwent urgent surgery, with persistent infection as the primary indication (73%). This distribution is consistent with the RISK-E (51%) and APORTEI cohorts, but differs substantially from EuroSCORE II, where elective procedures predominated (76.7% (7, 8). This mismatch likely contributed to EuroSCORE II's suboptimal calibration in our cohort.

### Discrimination

RISK-E demonstrated the highest discrimination (C = 0.741, 95% CI 0.61–0.85), followed by EuroSCORE II (C = 0.718, 95% CI 0.58–0.83) and APORTEI (C = 0.676, 95% CI 0.53–0.80). These results are broadly consistent with Varela et al., who reported RISK-E and EuroSCORE II with AUCs of 0.76 (0.69–0.82) and 0.74 (0.66–0.79), respectively, and APORTEI with AUC 0.75 (0.68–0.82), superior to our finding for APORTEI (15). Urso et al. reported notably higher APORTEI discrimination (AUC 0.88 ± 0.05) (16); however, their cohort had a lower proportion of urgent surgeries (28.8%), more closely resembling the APORTEI development population (36.1%), which may explain the divergence from our findings.

The modest C-statistics observed across all three scores (0.68–0.74) reflect the inherent difficulty of predicting surgical mortality in IE, where outcome is influenced by intraoperative findings, microbiological response, and postoperative complications not captured by preoperative variables alone (17).

### Calibration and clinical implications

A notable finding of this study is that all three scores systematically underestimated in-hospital mortality, as evidenced by positive calibration intercepts ranging from 1.14 to 1.75. APORTEI showed the greatest miscalibration (intercept 1.75, slope 1.425), while RISK-E demonstrated the closest agreement between predicted and observed risk (slope 0.962). The deviation in APORTEI's slope is likely attributable to the categorized collection of its component variables in this registry, which attenuates individual predictor weighting, as described in the Methods section.

The systematic underestimation of mortality across all scores has direct clinical implications. A surgeon relying on any of these tools in a similar resource-constrained setting would consistently receive risk estimates lower than the patient's actual probability of death, potentially influencing the timing and indication for surgery. Recalibration of existing scores, or development of locally derived prediction models, should be considered a research priority for IE surgical programs in Latin American tertiary centers.

### Limitations

This study has several limitations. Its retrospective single-center design limits generalizability and introduces potential information bias. The cohort comprised 70 patients with only 21 in-hospital deaths, limiting the precision of performance estimates. The wide 95% confidence intervals around all C-statistics (spanning approximately 0.12–0.14 AUC units) and the absence of statistically significant differences on pairwise DeLong testing preclude definitive conclusions regarding comparative model performance. Calibration estimates derived from a cohort with a limited number of outcome events should likewise be interpreted with appropriate caution. Data were collected over an eleven-year period, during which surgical techniques, antimicrobial regimens, and patient profiles may have evolved, introducing heterogeneity in management. APORTEI variables were collected in categorized form, which likely affected calibration. Post-discharge outcomes were not available, preventing assessment of 30-day or 90-day mortality. Finally, this study included only patients who underwent surgery; the excluded non-surgical IE population may differ systematically, limiting the scope of these findings to the surgical subgroup.

## Conclusions

In this retrospective cohort of patients undergoing cardiac surgery for IE at a Colombian tertiary-care center, RISK-E showed numerically superior discrimination (C = 0.741, 95% CI 0.61–0.85) and a calibration slope closest to unity (0.962); however, pairwise DeLong's tests showed no statistically significant difference in AUC between any two scores, and these numerical findings must be interpreted in the context of the limited sample size (70 patients, 21 events). EuroSCORE II demonstrated comparable discrimination (C = 0.718, 95% CI 0.58–0.83) despite not having been developed for IE-specific populations, while APORTEI exhibited the lowest calibration accuracy in this cohort, likely influenced by the categorized collection of its component variables. Notably, all three scores systematically underestimated in-hospital mortality, suggesting that their direct application in similar resource-constrained settings may lead to consistent underestimation of surgical risk. These findings highlight the need for external validation and recalibration of existing IE risk scores in Latin American populations and support the development of locally derived prediction models as a research priority for IE surgical programs in middle-income countries.

## Data Availability

The raw data supporting the conclusions of this article will be made available by the authors, without undue reservation.
